# Cancer Stem Cells and Pediatric Solid Tumors

**DOI:** 10.3390/cancers3010298

**Published:** 2011-01-14

**Authors:** Gregory K. Friedman, G. Yancey Gillespie

**Affiliations:** 1 Division of Pediatric Hematology and Oncology, University of Alabama at Birmingham, Birmingham, AL 35233, USA; 2 Department of Surgery, Division of Neurosurgery, University of Alabama at Birmingham, Birmingham, AL 35233, USA; E-Mail: yancey@uab.edu

**Keywords:** cancer stem cells, pediatrics, CD133, cancer initiating cells, tumor stem cells, tumor initiating cells, cancer progenitor cells, tumor progenitor cells, children

## Abstract

Recently, a subpopulation of cells, termed tumor-initiating cells or tumor stem cells (TSC), has been identified in many different types of solid tumors. These TSC, which are typically more resistant to chemotherapy and radiation compared to other tumor cells, have properties similar to normal stem cells including multipotency and the ability to self-renew, proliferate, and maintain the neoplastic clone. Much of the research on TSC has focused on adult cancers. With considerable differences in tumor biology between adult and pediatric cancers, there may be significant differences in the presence, function and behavior of TSC in pediatric malignancies. We discuss what is currently known about pediatric solid TSC with specific focus on TSC markers, tumor microenvironment, signaling pathways, therapeutic resistance and potential future therapies to target pediatric TSC.

## 1. Introduction

The axiom that “children are not little adults” has long been applied to the management of pediatric patients, and it certainly applies to cancer patients. Most pediatric cancers differ quite significantly from adult cancers in several important ways including: (1) incidence; (2) underlying etiopathogenesis; (3) response rates; (4) outcomes and (5) biology. Unlike adult cancers, childhood cancer remains a rare event, affecting approximately 15 per 100,000 children annually [1]. Whereas adult cancers are often associated with specific risk factors, the cause of most childhood cancers is unknown. Pediatric cancers typically respond better to current therapies than adult cancers, and through cooperative group efforts, the overall cure rate of childhood cancer has reached approximately 75%, which is much higher than adult cure rates [1-2]. The most important factor that helps explain these differences is tumor biology. Adult tumors are most commonly carcinomas that are derived from highly differentiated epithelial tissues exemplified in lung, breast, prostate and colon cancer. These cancer types and locations are almost never seen in childhood; in contrast, pediatric cancers are mainly embryonic in origin and are generally derived from non-ectodermal embryonal tissues. The most common pediatric solid tumors include brain tumors (25%), lymphomas (10%), neuroblastoma (8%), Wilms tumor (6%), and bone tumors (5%) [1].

Recently, a subpopulation of cells, termed ‘cancer stem cells’ or ‘tumor stem cells (TSC)’, has been discovered in many different adult tumor types including breast, brain, lung, prostate, melanoma, pancreas, colon, liver, head and neck and ovarian cancers [3-12]. These TSC have “stem cell” properties including multipotency, the ability to self renew, proliferate and maintain the neoplastic clone, and these cells are generally resistant to conventional chemotherapy and radiotherapy [13,14]. Therapies that are variously targeted to TSC markers, specialized niches, signaling and immunologic pathways, and other TSC mechanisms of resistance are currently being explored [15-17]. Additional therapies are being designed with the intent to reverse mechanisms of TSC resistance or to differentiate primitive TSC into more easily killed cancer cells [18]. Given the considerable differences in tumor biology between adult and pediatric cancers, the initial question is whether or not TSC exist and, if so, do they function similarly in pediatric malignancies as in adult tumors. To further confound the field, it is probably under-appreciated the extent to which normal tissue “stem cells” migrate to the site of a tumor and contribute to the tumor mass and to tumor progression [19]. This information would provide a rational basis to determine whether TSC can be targeted in a comparable fashion being developed for adult TSC. This review will focus on what is currently known about pediatric solid TSC and the targets they present for developing more effective therapies for pediatric solid tumors.

## 2. Tumor Markers

One of the main challenges in TSC research is distinguishing tumor cells from TSC. Determining TSC markers is crucial in order to study TSC and develop targeted therapies against them. Putative TSC have been identified by a variety of markers such as cell-surface proteins, nuclear or cytoplasmic proteins, transcription factors, enzymes and/or functional attributes. The most common markers used, with varying degrees of success, to identify TSC in adult tumors include CD133, CD44, CD24, CD90, CD34, CD117, CD20, side population (SP) (ability to exclude Hoechst dye), and aldehyde dehydrogenase 1 (ALDH1) [20]. The adult TSC markers characteristically specify a subpopulation of cells in a tumor that has a greater proliferative ability, is capable of maintaining the tumor after serially transplantation over several generations, and/or is more resistant to radiation and common chemotherapeutic agents. A fundamental limitation of this approach is that not all cells that express a given biomarker have functional attributes of TSC and, conversely, cells that lack detectable expression of a TSC marker may behave like TSC [21]. Current research is underway to identify improved markers or sets of markers that can identify a pure TSC population.

**Figure 1.**
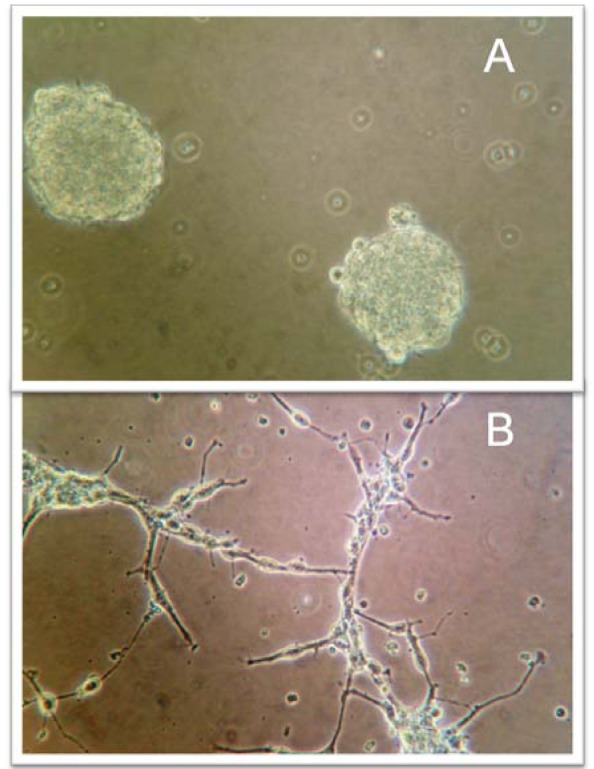
(**A**) Glioblastoma multiforme cells grown as neurospheres in serum-free medium supplemented with epidermal growth factor and basic fibroblast growth factor. (**B**) Cells grown in DMEM with fetal bovine serum and L-glutamine.

The most widely used cell surface molecule relied upon as a TSC marker is CD133 or prominin-1, a transmembrane protein with uncertain biological function that was initially discovered on hematopoietic stem and progenitor cells. CD133 has been utilized in a wide range of adult tumors and has become the most common marker used to identify pediatric TSC (Table 1) [20,22,23]. The first studies to utilize CD133 as a pediatric TSC marker occurred in brain tumors. Singh *et al.* purified CD133+ medulloblastoma (MB) TSC from patients’ tumors based on several functional criteria including a marked capacity for proliferation, a propensity for self-renewal, and capacity for asymmetric differentiation [24]. As a validation of the importance of TSC, they found that the self-renewal ability of the brain TSC was greatest in the most aggressive clinical samples of MB as compared with low-grade gliomas. Similarly, Hemmati *et al.* identified brain TSC in tumor samples from pediatric patients ranging in age from 15 months to six years who had MB, anaplastic astrocytoma, or glioblastoma multiforme (GBM) [25]. When cultured using stringent conditions in specially-formulated serum-free tissue culture medium with epidermal growth factor and basic fibroblast growth factor, tumor cells grew non-adherently in clumps of cells rather than as monolayers and cells in these tumor-derived “neurospheres” (Figure 1) expressed genes characteristic of neural stem cells including CD133, the transcription factor Sox2, and nuclear and cytoplasmic proteins musashi-1and bmi-1. More recent studies have used CD133 alone or in combination with nestin, an intermediate filament protein expressed in embryonic neuroglial cells, to isolate TSC in MB, to establish an anaplastic MB cell line with stem cell features, and to develop clinically relevant xenograft mouse models of MB and high-grade glioma [26-28]. CD133+ TSC have been identified in other pediatric brain tumors including ependymoma and atypical teratoid/rhabdoid tumor (AT/RT) [29-31]. The cell of origin of ependymomas may be the radial glia cells as tumor-derived spheres displayed an immunophenotype (CD133+, nestin+, radial glia marker RC2+, and brain-lipid binding protein (BLBP+)) similar to that of normal radial glia cells [29]. However, as will be detailed below, CD133 may not necessarily be the most accurate marker for tumor cells that display the functional characteristics that have come to be associated with TSC, and recently, several groups have suggested that CD15 (stage specific embryonic antigen 1 or SSEA-1), which is expressed on neural progenitor and stem cells, may be a better marker than CD133 of tumor-initiating cells in MB, glioma, and ependymoma [32-35].

After the discovery of CD133+ pediatric brain TSC, many investigators began examining the utility of CD133 as a TSC marker in a wide variety of other pediatric solid tumors including retinoblastoma, neuroblastoma, malignant melanoma, and renal tumors. Some of the earliest studies identified retinoblastoma stem-cell like cells that expressed embryonic, neuronal and retinal development related genes and markers including CD133 [36-38]. A more recent report by Balla *et al.* suggests that CD44, a cell surface glycoprotein involved in a wide variety of cell functions including adhesion and migration, and not CD133, may mark retinoblastoma stem-like cells [39]. CD44 has previously been implicated as a pancreatic and breast cancer TSC biomarker [40,41]. CD133+ cells that form “tumorspheres” were discovered in some human neuroblastoma cell lines and several cell lines could be induced into multilineage differentiation [42]. Importantly, CD133 expression in patient neuroblastoma and ganglioneuroblastoma samples increased significantly with the grade of the tumor and negatively correlated with patient survival time [43]. The authors suggested that CD133 may correlate with development and progression of neuroblastoma and may serve as an important indicator of prognosis. Similarly, Al Dhaybi *et al.* found CD133 expression seemed to correlate with aggressiveness and metastasis in childhood malignant melanoma [44]. In malignant rhabdoid tumor of the kidney (MRTK), a very aggressive malignancy in infants, Yanagisawa *et al.* found that as few as 1,000 CD133+ MRTK cells were able to initiate tumors in NOD/scid mice, yet the metastatic potential of these cells was unaffected compared to CD133- cells, leading the authors to conclude that CD133+ cells may determine metastatic fate of MRTK cells and CD133- may support tumor progression and metastasis [45]. In an evaluation of putative stem cell markers in Wilms’ tumor xenografts, CD133 and neural cell adhesion molecule (NCAM or CD56), which can be found on developing renal tubules and in kidneys recovering from ischemic injury, were felt to most likely contain the stem cell fraction, however under clonogenicity assays, only NCAM+ cells were highly clonogenic and overexpressed the Wilms tumor “stemness” genes along with the clinically bad prognostic marker, TOP2A [46,47].

Pediatric sarcomas are another group of tumors where CD133 has been used as a marker to identify tumor stem cells. Tirino *et al.* [48] first identified CD133+ cells in osteosarcoma with stem-like features including the ability to form tumor spheres, high proliferation rate, cell cycle detection in the G2/M phase, positivity for the nuclear protein Ki-67 which is associated with cellular proliferation, SP profile and expression of ATP-binding cassette (ABC) transporters which have been implicated in chemotherapy drug resistance. In addition to CD133, nestin, the transcription factors Oct3/4 and Nanog, which are critically important for the self-renewal of undifferentiated embryonic stem cells, have been detected in patient osteosarcoma tumor tissue or cell lines suggesting that there are several potential markers of osteosarcoma stem cells [49-51]. Other groups have been trying to uncover the cell of origin for rhabdomyosarcoma (RMS) and Ewing's sarcoma (EWS). Studies have suggested either mesenchymal stem cells (MSC) or muscle satellite cells may be the cell of origin for RMS and MSC for EWS [52-56]. Our group has discovered a myogenically primitive RMS cell that has stem cell features and expresses CD133 [57]. Similarly, Suva *et al.* found CD133 to mark EWS cancer stem cells with properties similar to MSC [58]. The CD133+ EWS cells were able to differentiate along adipogenic, osteogenic and chondrogenic lineages like MSC. Other types of non-rhabdomyosarcoma soft-tissue sarcoma such as synovial sarcoma may have cells that express CD133 and behave like TSCs [59].

While CD133 has proven useful in identifying tumor cells with stem-like features, the marker is far from perfect. The implied assumption is that a cluster of differentiation marker should be associated with a defined cell type with defined functional attributes as in the familiar CD4 and CD8 T lymphocyte subpopulations. However, defining and isolating CD133+ and CD133- populations can be difficult, since expression levels are on a continuum and not qualitatively associated with tumor cells, and expression is transilient. This may partially explain why several studies in adult cancers including colon, lung and brain have demonstrated that CD133- tumor cells can initiate tumors and why both CD133+ and CD133- DAOY MB cells displayed equivalent stem-like frequencies [60-65]. Moreover, CD133 was proven to be a marker of bioenergetic stress in human gliomas with the CD133+ percentage capable of increasing dramatically under hypoxic conditions [66]. CD133 expression is further confounded by the recent observation that HIF-1 activation drives increased CD133 expression under hypoxia [67, 68]. Whether this increased population of CD133+ cells represents a tumor-initiating population and a resistant population of cells has not been established. Several chemoresistant markers, TIMP-1 and LAMP-1, were upregulated along with CD133 expression in glioblastoma cell line-derived spheroids under hypoxia compared to normoxia suggesting that the increased population of cells may be clinically relevant in some tumor types [68]. Further studies are needed to determine the relevance of CD133- cells and the role of hypoxia and CD133 expression in pediatric tumors.

Another limitation of many of the studies attempting to define a pediatric TSC based on CD133 expression is the use of sphere formation (e.g., neurosphere, sarcosphere) to confirm the presence of TSC and the proliferative capacity of CD133+ cells. This assay is problematic for several reasons. When performed in a liquid medium as opposed to a semisolid medium, neurospheres can form overnight from a disaggregated suspension of single cells by a re-aggregation process and these structures are also prone to combine with other spheres, thus making clonality difficult to confirm [69]. Like others, we have found that both CD133+ and CD133- tumor cells can form spheres; sphere generation appears to be a cell culture artifact due to serum free medium use and does not have an *in vivo* parallel [70,71]. Importantly, not all the cells within a sphere are undifferentiated TSC, and in fact, the majority of cells may be nonstem cells. Interestingly, addition of laminin to serum-free medium used for TSC culture will convert many neurospheres to monolayers of cells adherent to the culture substratum. This phenomenon may suggest a critical role of the microenvironment in promoting the survival of TSC [72]. Newer methods for studying TSC must be developed to improve our understanding of these cells.

The most common other method used to distinguish potential pediatric TSC is sorting cells based on selection of Hoechst 33342 dye-excluding SP cells. This technique relies on a cell's ability to extrude Hoechst 33342 dye as measured by flow cytometry. The process by which the dye is excluded involves various members of the ABC transporters family which play a major role in drug resistance mechanisms. The SP tends to be a small minority of the entire population of tumor cells but enriched in TSC [73]. Whether these are actual TSC is unclear, however, the SP is thought to be important due to the inherent chemotherapeutic resistance of these cells. The first study confirming the presence of a SP in pediatric tumors examined seven neuroblastoma, four rhabdomyosarcoma and five Ewing's sarcoma cell lines [74]. All but one cell line (Ewing's sarcoma) contained a SP ranging from 0.12% to 14.6%. Similarly, in separate studies, several osteosarcoma cell lines were found to have SP cells that were capable of forming spherical colonies and inducing tumorigenesis, and a SP from a hepatoblastoma cell line was able to form tumors in mice whereas tumors did not form in the non-SP [75,76]. In each of these studies, the resistance patterns of the SP were not tested. The one pediatric study examining the resistance of SP involved three neuroblastoma cell lines derived from patients at the time of initial presentation and again at relapse after multiple chemotherapeutics [77]. The investigators discovered a higher SP in the relapsed cell lines as compared to the pretreatment lines, and the relapsed lines formed colonies more efficiently and had an increased proliferative ability. In addition, expression of stem cell genes Nanog and Oct3/4 were higher in the relapsed SP suggesting that the SP represented a tumor stem-like fraction that was resistant to conventional chemotherapy. Further studies are needed to confirm the role of SP in pediatric tumors and the relationship of SP with other putative TSC markers such as CD133.

## 3. Tumor Microenvironment

**Figure 2.**
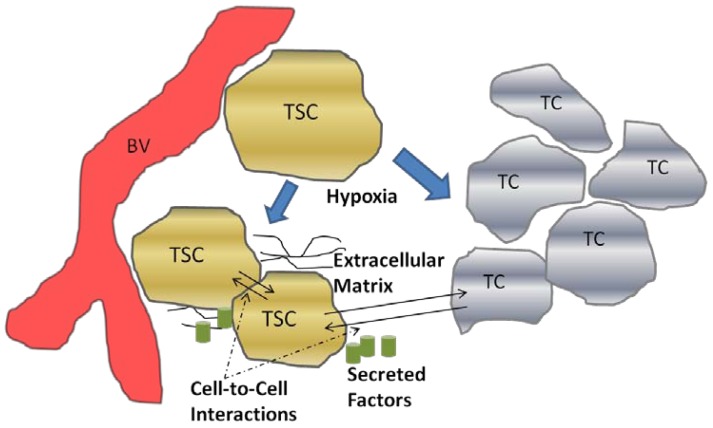
Tumor stem cell (TSC) niche. TSCs give rise to tumor cells (TC) and other TSCs. TSCs are thought to undergo self-renewal near blood vessels (BV) in the TSC niche. Multiple factors contribute to the microenvironment including hypoxia, the extracellular matrix, cell-to-cell interactions, secreted factors and signals, and the tissue makeup itself.

With the discovery of TSC, there has been an increased focus on understanding the specialized microenvironment or “niche” where TSC reside and are regulated and maintained. This niche includes cell to cell interactions, the extracellular matrix, secreted factors and signals, the tissue makeup and limitations it poses, and oxygen tension (Figure 2) [78,79]. How these factors combine to promote TSC self-renewal and tumor cell proliferation is poorly understood. Much of what is known regarding the TSC niche has been garnered from the normal stem cell niche. In normal tissues, the stem cell niche plays a critical role in stem cell maintenance through a tight balance of inhibiting and promoting factors which maintain homeostasis or can shift stem cells towards proliferation and differentiation [80]. TSC may arise from a disruption in the balance with a resultant swing towards proliferation.

Several studies have demonstrated the importance of the microenvironment's effect on pediatric TSC and an invasive tumor phenotype. Matrix metalloproteinases (MMPs) are zinc-dependent endopeptidases that degrade the extracellular matrix and have been implicated in tumor growth, angiogenesis, invasion, migration and metastasis [81]. They are thought to mediate many of the changes in the microenvironment responsible for tumor progression [82]. Annabi *et al.* examined the role of membrane type-1 (MT1) MMP and other MMPs in the regulation of CD133+ MB DAOY TSC [83]. They found a correlation between expression of vascular endothelial growth factor (VEGF) and basic fibroblast growth factor and increasing CD133 expression. When DAOY cells were induced to form neurospheres, gene expression of CD133, MT1-MMP and MMP-9 were induced and resulted in increased neurosphere invasiveness. When MT1-MMP and MMP-9 genes were silenced, neurosphere generation and cell invasiveness was decreased. Similarly, TSC from malignant gliomas including a pediatric GBM xenograft secreted markedly elevated levels of VEGF which was further induced by hypoxia, and conditioned medium from the TSC increased endothelial cell migration *in vitro* [84]. Further evidence of hypoxia's role in maintaining the TSC niche was seen in neuroblastoma and rhabdomyosarcoma cell lines [85]. Researchers found a highly tumorigenic SP of cells was localized in the hypoxic zones *in vivo* and that hypoxia increased the SP fraction suggesting that hypoxia plays an important role in the TSC niche. Taken together, these studies highlight the significance of the TSC niche in pediatric tumors. By gaining a better understanding of this niche, researchers will be able to determine factors that regulate TSC and perhaps develop new targets to eradicate these cells.

## 4. Signaling Pathways

**Figure 3.**
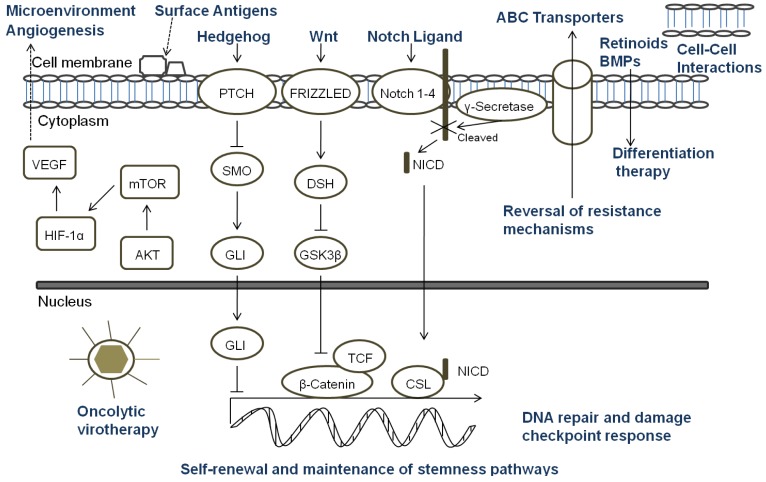
Potential strategies for targeting tumor stem cells (TSC) include directed attacks at surface antigens, altering the microenvironment and cell-to-cell interactions, inhibiting angiogenesis, targeting embryonic signaling and self-renewal pathways, reversing resistance mechanisms such as ABC transporters, differentiating primitive TSC into more susceptible tumor cells, inhibiting DNA repair, or targeting TSC in unique, cell cycle independent ways like oncolytic virotherapy.

A key aspect of the TSC niche is the balance of signals received, and over recent years considerable attention has been directed towards understanding the role of signaling pathways, which are critical mediators of normal stem cell biology, in cancers. The embryonic signaling pathways most commonly implicated in tumorigenesis include Hedgehog, Notch, and Wnt pathways (Figure 3). Sonic Hedgehog (SHH) signaling is important in embryonic cell development and proliferation and aberrant pathway activation can lead to tumor formation, tumor cell self-renewal and the development of metastatic disease [86,87]. Similarly, Notch plays a crucial role in biological functions of development and cell fate including cell differentiation and proliferation [88]. Constitutive activation of Notch can lead to tumorigenesis and cell survival, and Notch activity is involved in tumor angiogenesis [89]. The Wnt family proteins help direct a wide range of developmental processes including cell fate, proliferation, motility, and polarity [90]. Wnt is also a critical regulator of normal stem cells and cell homeostasis [91]. Dysregulation of the Wnt pathways has been implicated in tumor formation, proliferation, and maintenance. Due to the many similarities between the role of the SHH, Notch, and Wnt pathways and TSC in tumor development, maintenance, and progression, recent research has begun to focus on the intimate relationship of embryonic signaling pathways and TSC. Table 2 highlights the pediatric malignancies including TSC that have been linked to SHH, Notch, and/or Wnt pathways [92-94].

All of the current pediatric studies demonstrating that progenitor and stem cells can respond to embryonic signaling have been in MB or primitive neuroectodermal tumors (PNET). Aberrant SHH signaling has been implicated in MB, and recently was used to define one of four distinct molecular variants of MB [95]. An earlier study found that deleting *Patched* gene, an antagonist of SHH, in mouse multipotent stem cells led to the expansion of the stem cell population and growth of the granule neuron precursors derived from the stem cells resulting in rapid tumor formation with 100% of the animals dying from MB by four weeks of age [96]. When SHH was inhibited with cyclopamine, viability and tumorigenic potential was reduced in MB and PNET cell lines [97]. Cells which expressed CD133 were more resistant to cyclopamine inhibition suggesting a potential mechanism of CD133+ TSC treatment resistance. In another study, Notch signaling levels were higher in the stem cell fraction of MB cells, and Notch activation blockade resulted in a viable population of more differentiated cells that continued to grow but were unable to form soft-agar colonies or tumor xenografts [98]. The remaining population of cells after the blockade had nearly five-fold less CD133+ cells and no SP suggesting that the Notch signal pathway is critical to MB stem cells. Understanding the role of embryonic signaling pathways in TSC is still a nascent area of research, but these initial studies suggest further investigation would be worthwhile.

## 5. Therapeutic Resistance

While there have been differences in opinion regarding the “stemness” of TSC, the therapeutic resistance of TSC to current treatment modalities such as chemotherapy and radiation make these cells clinically relevant irrespective of their origin. Putative mechanisms of chemotherapy and radiotherapy resistance in pediatric TSC are summarized in Table 3. Resistance to chemotherapeutic agents has been demonstrated in neuroblastoma stem cells and sarcoma stem cells including Ewing's sarcoma and osteosarcoma. CD133+ neuroblastoma cells formed tumorspheres more efficiently than CD133- cells, and the tumorspheres were more resistant to doxorubicin than bulk cells. The tumorspheres showed a small increase in CD133 expression after treatment suggesting that the CD133+ cells were more resistant to the agent [42]. Similarly, an osteosarcoma and Ewing's sarcoma cell line grew in doxorubicin- and cisplatin- resistant sarcospheres which showed stem-like properties of self-renewal and increased expression of stem cell-related genes [99]. When two neuroblastoma cell lines were separated in CD133+ and CD133–fractions by magnetic microbeads and tested for chemosensitivity, the phosphorylated forms of both ERK and p38 kinases, which indicates activation and has been associated with cell survival mechanisms, were expressed at higher levels in CD133+ cells, and those cells were more resistant to commonly used treatment drugs including cisplatin, carboplatin, doxorubicin and etoposide [100]. A comparable study in Ewing's sarcoma tumors and cell lines found that CD133+ cells were low or absent in most tumors, and the CD133+ fraction was more resistant to chemotherapy in only some of the tumors [101]. The low percentage of CD133+ cells in the tumor samples may have contributed to the inconsistent response to chemotherapy.

Recently, Hussein *et al.* showed that brain tumor xenografts, including ependymoma, GBM, and MB, grown as neurospheres were more resistant to etoposide than cells grown as a monolayer [102]. The monolayers had increased DNA damage compared to the neurospheres, which repaired the DNA damage faster. ABC transporter proteins were enriched in the MB TSC population with CD133+ cells containing twice as many transporters as CD133- cells. Since most chemotherapy targets cell cycling and brain TSC may have better control of their replicative response by becoming intermittently quiescent, the authors compared the cell cycles of xenografts grown as neurospheres or as monolayers. While neurosphere cells were seen in all phases of the cell cycle, they had an increased percentage of cells in G_0_/G_1_ and a decrease in S phase and G_2_/M compared to cells growing as monolayers suggesting that quiescence may play a role in TSC resistance to chemotherapy.

Radioresistance of pediatric TSC has been demonstrated in three brain tumor types: GBM, AT/RT, and MB. Using a pediatric GBM xenograft, Bao *et al.* found that CD133+ cells survived ionizing radiation and repair DNA damage better than other tumor cells by preferentially activating the DNA damage checkpoint [103]. Activated phosphorylation of the checkpoint proteins ATM, Rad17, Chk1 and Chk2 were significantly higher in the CD133+ fraction, and relative radioresistance of the CD133+ cells was reversible with the pharmacologic inhibition of Chk1 and Chk2. Similarly, CD133+ AT/RT cells and MB spheroids were able to more effectively resist irradiation than other tumor cells with TNF-related apopotosis-inducing ligand [31,104]. An upregulation of anti-apoptopic genes was found in both studies. Due to the resistance of TSC to conventional therapeutics like radiation and current chemotherapy and their role in tumor maintenance, recurrence, and metastasis, targeted therapies must be developed.

## 6. Targeted Therapy

Since standard therapies are directed primarily at bulk tumor cells, the challenge that researchers face is to design therapies that specifically seek out and target TSC. Options for targeting TSC (Figure 3) include any aspect of the cells which make them unique: specific markers, the TSC niche, or unique signaling pathways. Other potential ways to target TSC involve mechanisms of attack that differ from conventional therapeutics, reversal of resistance mechanisms, or differentiation therapy [18]. For a surface marker to be a useful target, that marker must identify all of the TSC in a particular tumor and must be a unique marker not found on normal cells. In pediatric TSC, currently there are a limited number of markers used to identify these cells. CD133 is the most common marker used, however it may not mark the entire stem cell population, and its expression is not always stable with markedly increased expression seen under hypoxic conditions [66-68]. Moreover, CD133 is found on the surface of normal stem cells including hematopoietic, endothelial, and neural stem cells [22,23]. Thus, biomarkers that are more specific for solid tumor cells should be sought which then could be the focus of targeted therapies involving the immune system or small molecule inhibitors.

The TSC niche which maintains and directs the activity of TSC provides a potential therapeutic target. Identifying the components of the microenvironment that are responsible for TSC self-renewal, proliferation, differentiation, and quiescence is a critical step in the development of directed therapies, and currently this area of research is in its infancy and poses its own set of challenges [105]. The TSC niche must be distinct from the normal stem cell microenvironment or a potential therapeutic may damage essential cells for normal tissue maintenance and repair. If TSC reside in multiple areas of a tumor, the niche must be the same in each of those areas. It is possible that there are multiple niches, and a targeted therapy must be able to attack all of the microenvironments to eradicate TSC. Additionally, the niche must be the same for primary tumor sites and metastatic sites. The possibility exists that there are stage-specific TSC which have unique roles and microenvironments depending on the stage of tumor progression. The only report of targeting the TSC niche in pediatric solid tumors to date was in a pediatric GBM xenograft. The anti-VEGF antibody bevacizumab eliminated the proangiogenic effects of glioma TSC on endothelial cells [84]. Moreover, the agent suppressed growth of tumors derived from glioma TSC in mice suggesting that targeting the TSC microenvironment may provide a critical alternative treatment.

Directed therapy at the embryonic signaling pathways SHH, Notch and Wnt, which have been shown to be aberrant in pediatric TSC and are important part of the TSC niche, is another promising approach [106-109]. Successful targeting of these pathways necessitates a comprehensive grasp of the regulation of each pathway and their interaction with other pertinent pathways since SHH, Notch and Wnt are important to normal stem cell populations as well [86,88,90]. Currently, no pediatric studies have focused specifically on the effect of targeting these pathways in TSC; consequently this is a relatively unexplored and promising area for future pediatric research.

**Figure 4:**
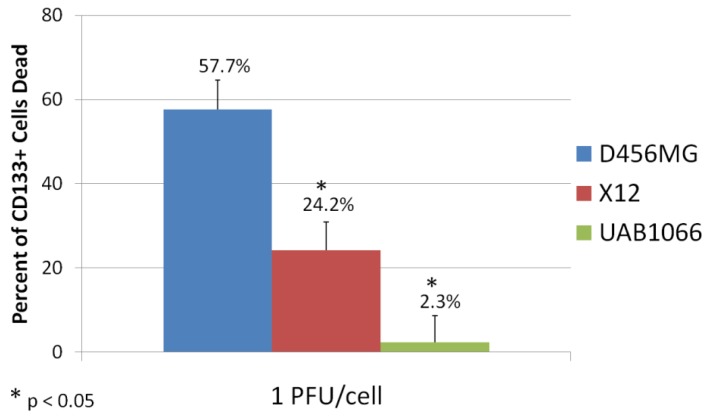
The CD133+ glioma stem cells in the pediatric GBM xenograft D456MG were significantly more sensitive to killing than several adult GBM xenografts tested after 72 hours of low dose infection (1 plaque-forming unit (PFU) per cell) with G207, an engineered herpes simplex virus used in adult GBM trials at the University of Alabama at Birmingham.

Another potential method to eradicate pediatric TSC is utilizing mechanisms of cell killing that differ from current conventional therapies. Oncolytic virotherapy is one of these novel approaches. Oncolytic viruses typically work in two main ways: as a direct, targeted attack by containing mutations that cause the virus to spare normal cells but infect tumor cells that then die and release infectious virus to neighboring tumor cells, or by expressing therapeutic foreign gene products that either directly or indirectly lead to cell death. The potential therapeutic benefit of virotherapy in treating pediatric malignancies and the effects of virotherapy on TSC, including pediatric tumors, have been studied in a number of viruses including herpes simplex virus (HSV), adenovirus, myxoma virus, reovirus, and vesicular stomatitis virus [110,111]. We found that the TSC from a pediatric GBM xenograft were more sensitive to killing by engineered HSV than several adult GBM tested (Figure 4) [112]. Cripe's lab showed that an engineered HSV effectively targeted and killed chemoresistant CD133+ neuroblastoma cells [42]. These studies suggest oncolytic virotherapy may be a useful alternative approach to kill resilient TSC. Reversing TSC resistance mechanism by blocking ABC transporters that efflux cytotoxic drugs, for example, or promoting differentiation of TSC to more susceptible tumor cells are additional alternative strategies that have not been specifically examined in pediatric solid tumors and require further study [18].

## 7. Conclusions

TSC have only recently been discovered in a wide variety of pediatric solid tumors and the current understanding of pediatric TSC identity, function, microenvironment, and resistance patterns is quite primitive. Better TSC markers are needed, so that all TSC in a tumor population can be identified and their role in tumor formation, maintenance and metastasis can be elucidated. Once improved markers are identified, further study into the TSC niche such as aberrant signaling pathways is imperative to understand how TSC interrelate with neighboring cells and other tumor cells. Research must not only focus on adult TSC but also pediatric TSC because tumor biology is quite different between adult and pediatric tumors, and therefore stark differences may be discovered in the presence, function and behavior of pediatric TSC. Once there is a greater understanding of pediatric TSC, novel, targeted therapies can be developed to help eradicate these resilient cells, and hopefully improve outcomes for children with difficult to treat or relapsed solid tumors.

## Figures and Tables

**Table 1.** Markers used to define tumor stem cells (TSC) in pediatric cancers.

**Malignancy**	**Cell Surface Proteins**	**Nuclear/Cytoplasmic Proteins**	**Transcription Factors**	**Functional/Enzymes**	**Ref.**
AT/RT	CD133				[31]
Ependymoma	CD133, CD15	Nestin, BLBP, RC2			[29,30,32]
Ewing's Sarcoma/PNET	CD133			SP	[58,74]
Glioma	CD133, CD15	Musashi-1, bmi-1	Sox-2		[24,25,32]
Hepatoblastoma				SP	[76]
Malignant rhabdoid tumor of the kidney	CD133				[45]
Medulloblastoma	CD133, CD15	Nestin		SP	[24-28,32-34]
Melanoma, childhood	CD133				[44]
Neuroblastoma	CD133	Nestin		SP	[42,43,74,77]
Osteosarcoma	CD133,	Nestin	Oct3/4, Nanog	SP	[48-51,75]
Retinoblastoma	CD133, CD44	Nestin, musashi-1, bmi-1	Oct3/4, Nanog, pax-6, chx10	ALDH1, SP	[36-39]
Rhabdomyosarcoma	CD133			SP	[57,74]
Wilms Tumor	CD133, NCAM				[46]

**Table 2.** Pediatric solid tumors and tumor stem cells (TSC) associated with aberrant embryonic signaling pathways. MB, medulloblastoma; PNET, primitive neuroectodermal tumors.

**Signal Pathway**	**Associated Malignancies**	**TSC**	**Ref.**
Sonic Hedgehog	Ependymoma, hepatoblastoma, MB, neuroblastoma, rhabdomyosarcoma, Wilms' tumor	MB, PNET	[93-97]
Notch	Ependymoma, MB, neuroblastoma, osteosarcoma	MB	[92,98]
Wnt	Ependymoma, hepatoblastoma, MB, neuroblastoma, PNET, Wilms' tumor		[94]

**Table 3.** Putative mechanisms of chemoresistance and radioresistance of pediatric tumor stem cells (TSC). MB, medulloblastoma.

**Chemoresistance Mechanism**	**Tumor Type**	**Ref.**
Efficient DNA repair ability	Ependymoma, Ewing's sarcoma, GBM, MB, osteosarcoma	[99,102]
Differential expression and phosphorylation of kinases	Neuroblastoma	[100]
Low Proliferative Ki-67 index	Childhood melanoma	[44]
ABC multidrug resistance	MB, neuroblastoma	[42,102]
Quiescence	Ependymoma, GBM, MB, PNET	[102]
**Radioresistance Mechanism**		
Preferential activation of DNA damage response	Glioblastoma	[103]
Upregulation of anti-apoptotic genes	MB, AT/RT	[31,104]
